# Chios Mastic Essential Oil in Sodium Alginate Edible Films Combined with High-Pressure Processing as *Listeria monocytogenes* Inhibitors in Cheese Slices

**DOI:** 10.3390/gels12030255

**Published:** 2026-03-18

**Authors:** Olga S. Papadopoulou, Anthoula A. Argyri, Eleftherios Kalogeridis, Konstantinos C. Mountzouris, Chrysoula C. Tassou, George-John Nychas, Nikos Chorianopoulos

**Affiliations:** 1 Institute of Technology of Agricultural Products, Hellenic Agricultural Organization-DIMITRA, S. Venizelou 1, 14123 Lycovrissi, Greece; anthi.argyri@elgo.gr (A.A.A.); ctassou@elgo.gr (C.C.T.); 2 Laboratory of Microbiology and Biotechnology of Foods, Department of Food Science and Human Nutrition, School of Food and Nutritional Sciences, Agricultural University of Athens, Iera Odos 75, 11855 Athens, Greece; leftokalog97@gmail.com (E.K.); gjn@aua.gr (G.-J.N.); nchorian@aua.gr (N.C.); 3 Laboratory of Nutritional Physiology and Feeding, Department of Animal Science, School of Animal Biosciences, Agricultural University of Athens, Iera Odos 75, 11855 Athens, Greece; kmountzouris@aua.gr

**Keywords:** *Listeria monocytogenes*, cheese, Chios mastic gum essential oil, high-pressure processing, antimicrobial packaging, ATR-FTIR

## Abstract

The antimicrobial effect of Chios mastic gum essential oil (mastic EO) was evaluated in vitro in a variety of spoilage and pathogenic bacteria and yeast strains isolated from spoiled cheeses with concentrations ranging from 0.006 to 2% (Minimum Inhibitory Concentration (MIC)) and in situ (cheese slices). The mastic EO (2%) was incorporated in sodium alginate edible gel films (Mastic Edible Films (MEFs)), and then the films were applied between the cheese slices that had been previously inoculated with a cocktail of three strains of *Listeria monocytogenes* (on both sides of the slices) and subjected or not to high-pressure processing (HPP). Cheese samples were vacuum-packaged and cold stored (4 °C), and microbiological, pH and organoleptic (in pathogen-free slices) analyses were employed, while Fourier Transform Infrared (FTIR) spectroscopy was applied as a rapid technique to monitor the biochemical changes present on the slices. Samples without MEF, without the pathogen and with or without HPP were employed as controls. Results showed that the MIC of the mastic EO varied from 0.01% to 1.8% depending on the species and/or strains. Pathogen’s growth was suppressed by HPP, MEF or their combination, which showed the highest efficacy. These results could provide useful data to support risk assessment studies on ready-to-eat foods. Finally, FTIR implementation with data analytics was found to be satisfactory, indicating FTIR’s potential as a reliable information source for cheese quality control.

## 1. Introduction

Semi-hard cheeses made from pasteurized milk are ready-to-eat (RTE) food products and are generally considered safe. However, post-processing contamination of cheeses with *Listeria monocytogenes* can take place either in the dairy processing environment or during further distribution of cheese products [1,2]. Particularly in deli retail stores where cheeses can be sold in cuts or sliced into consumer portions, post-processing contamination with the pathogen can occur during handling and storage [3]. In extended cold storage of RTE products, proliferation of the pathogen can occur, reaching high numbers and thus posing the risk of being ingested upon consumption [4]. The European Food Safety Authority reported that human invasive listeriosis is caused by consumption of RTE foods when pathogen concentration exceeds 4 log CFU/g, whereas 1/3 of these incidents are due to the growth of the pathogen in consumers’ households [5].

Various methods have been reported in the literature regarding food processing to ensure safety and delay spoilage. For example, high-pressure processing (HPP), which is a non-thermal treatment, is effective at extending the shelf life of various food commodities [6,7], at controlling spoilage and/or pathogenic microorganisms, and concurrently, the impact of HPP on the nutritional and sensory aspects of the product is minimal compared to typical thermal technologies [8]. The application of HPP (in different time/pressure combinations) to control *L. monocytogenes* after artificial inoculation (7 logs) of Gorgonzola cheese and during short cold storage was studied by Carminati et al. [9]. Also, the study of Tomasula et al. [10] examined the survival of five *L. monocytogenes* strains inoculated in Queso Fresco cheese after HPP and during two months of storage at 4 and 10 °C. In both studies, it was shown that higher pressures (>600 MPa) were more efficient in eliminating the pathogen below the detection threshold of the plating method; however, the pathogen managed to recover during storage [9,10]. An additional approach of combining HPP treatment with natural antimicrobials (thyme essential oil) to reduce the *L. monocytogenes* population was examined by Bleoancă et al. [6] in a fresh cheese. In this study, the pathogen was better inactivated by the combination of the two hurdles (HPP and thyme essential oil) applied to fresh cheese; however, the recovery of the pathogen during storage was not evaluated [6].

Essential oils (EOs) have also been used to control both spoilage and pathogenic bacteria; this is the case with Chios mastic gum EO, which derives from a small evergreen shrub named *Pistacia lentiscus* L. var. Chia (Family *Anacardiaceae*). Although mastic gum is already being used as a flavoring agent in foodstuffs or cosmetics, the potential antimicrobial properties of mastic EO in food have not been extensively studied. Some limitations of the employment of EOs in the food matrix are their intense odor, their high volatility and the possibility of toxicity in high concentrations [11]. The application of the EOs after encapsulation in edible films or coatings can mitigate those limitations and thus be considered one of the potential application areas of active food packaging [12,13]. In this sense, the EO can be incorporated into biopolymer ingredients, such as sodium alginate films, to avoid direct application to the food, thus minimizing the negative organoleptic effects [12,13,14].

The development of instrumental techniques to enable more objective, rapid, and less expensive assessments of cheese quality is of major interest because the present microbiological and sensory methods to determine cheese quality are time-consuming and expensive [15]. Fourier Transform Infrared (FTIR) spectroscopy is a rapid fingerprinting technique that, when combined with multivariate statistical tools, has aided considerably in the quantitative and qualitative detection of food spoilage in a variety of food commodities, such as dairy products, fresh meat, fish, etc. [16,17,18,19].

Thus, the purpose of this study was (i) to assess the antimicrobial impact of the mastic EO against several microorganisms (spoilage and pathogenic bacteria and yeasts) through the determination of the Minimum Inhibitory Concentration (MIC) of the EO in vitro using an optical density method, and (ii) to assess the efficacy of the applied hurdles (HPP and mastic EO incorporated in edible films) and their combination to control *Listeria monocytogenes* in cheese slices during cold storage under vacuum packaging. The different hurdles applied were the employment of HPP, the antimicrobial packaging with mastic EO encapsulated in sodium alginate edible films (MEFs), and their combination. In addition, the current study, besides the classical microbiological approach, applied a rapid non-invasive methodology based on FTIR-ATR analyses using a support vector machine (SVM) analysis to predict the microbial counts, classify the samples in freshness categories (fresh, semi-fresh and spoiled samples) and discriminate the samples based on the different hurdles applied (HPP, MEF and their combination).

## 2. Results and Discussion

### 2.1. Antimicrobial Assays In Vitro

The inhibition caused by the mastic EO in the population of *L. monocytogenes* is presented in Supplementary Figure S1. The data are presented after plotting the different EO concentrations on a logarithmic scale with the fractional area modeled by Lambert and Lambert [20]. The MIC values for each microorganism that were estimated after modeling the experimental data are presented in Supplementary Table S2. The EO inhibited yeast strains in concentrations less than 0.8%, while the inhibitory effect on most of the LAB strains was moderate (0.77–1.47%). In more detail, for most of the examined yeast strains (five out of eight strains tested), low concentrations of 0.01–0.02% (*v*/*v*) of EO were needed to inhibit their growth. In contrast, *L. monocytogenes* strains were inhibited by moderate and/or high concentrations, varying from 0.9 to 1.8% (*v*/*v*) EO, depending on the strain. Based on these findings, a three-strain cocktail of the pathogen, exhibiting varying degrees of inhibition by the EO, was selected for the in situ experiment. To ensure effective inhibition of *Listeria* in the more complex environment of a real food matrix, such as cheese, a higher concentration of 2% mastic EO was chosen.

### 2.2. Microbiological Analysis and pH Determination of the Cheese Slices

**Figure 1:**
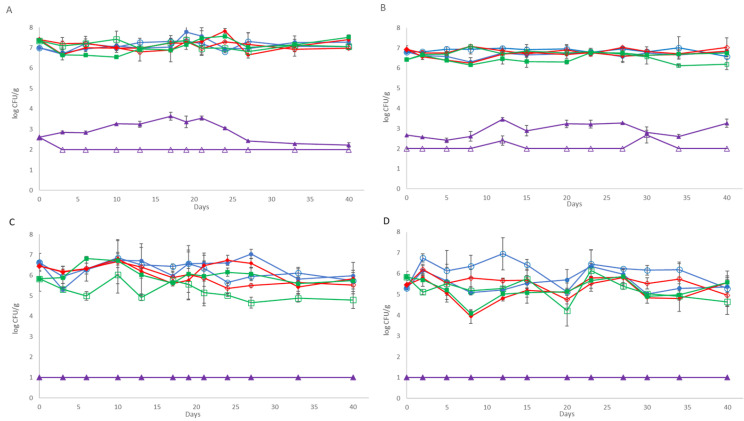
Population of TVC (●), mesophilic LAB (◆), lactococci (■) and yeast and moulds (▲) in control samples and population of TVC (○) mesophilic LAB (◇), lactococci (□) and yeast and moulds (△) in MEF samples during storage of cheese slices at 4 °C without HPP: (**A**) batch 1 and (**B**) batch 2, and with HPP: (**C**) batch 1 and (**D**) batch 2. The bars represent the mean values of three replicates ± standard deviations. (The population of TVC, mesophilic LAB and lactococci of control and HPP samples without MEF was also included in Papadopoulou et al. [21]).

The microbial populations of mesophilic LAB, lactococci, Total Viable Aerobic Counts (TVCs) and yeast and moulds of cheese samples during storage are shown in Figure 1. The microbiota of the control cheese samples (untreated) are presented in detail in the previous study by Papadopoulou et al. [21], since the experimentation regarding the control cases without the pathogen was the same as in that study. In brief, on day “0”, the population of mesophilic LAB and lactococci was found to be close to 7 log CFU/g in both batches. During storage of the control cheese samples, mesophilic LAB and lactococci were found to be the dominant microorganisms throughout storage, with population levels over 6.16 log CFU/g for both batches examined, since counts of TVC were found to be similar to those of LAB. Among the other tested microorganisms, only yeasts and moulds were detected in low population numbers at day 0, though their population was slightly higher in both batches by the end of storage (40 days), as was already described by Papadopoulou et al. [21].

With regard to the samples with MEF without the application of HPP, the mesophilic LAB, lactococci and TVC population at the beginning of storage were found to be 7.21 ± 0.30, 7.08 ± 0.24, 6.69 ± 0.29 log CFU/g and 6.75 ± 0.11, 6.67 ± 0.06, 6.81 ± 0.01 log CFU/g, respectively, for the two batches (Figure 1). However, in MEF samples during storage, the LAB population decreased by ca. 0.5 log CFU/g in comparison to the control samples. Specifically, the population of LAB varied between 6.90–7.33 log CFU/g (*p* ≥ 0.05) and 6.70–6.94 log CFU/g (*p* < 0.05) during storage at both batches, respectively, while the lactococci population was found to be slightly higher in the first batch (6.82–7.52 log CFU/g, *p* ≥ 0.05) and lower (5.70–6.80 log CFU/g, *p* ≥ 0.05) in the second batch compared to the control. In addition, counts of TVC were correlated with the counts of LAB and/or lactococci, which were the dominant microbiota depending on the different sampling days throughout storage. Among the examined spoilage microbiota, yeast and moulds were periodically detected only in the second batch (in one out of the three examined samples) at populations of 2.40 log CFU/g (day 10) and 2.68 log CFU/g (day 30). Thus, it was demonstrated that the addition of the MEF films may inhibit the growth of yeasts and moulds in comparison with the control samples, as these microorganisms were able to grow in control samples, reaching population levels of up to 3.26 log CFU/g by the end of storage. Overall, the differences between the examined microbial populations at MEF and control samples throughout cold storage were found to be significant (for mesophilic LAB and TVC, *p* < 0.05) only in the second batch. Enrichment methods confirmed the absence of *Salmonella* sp. and *L. monocytogenes* in the cheese samples.

The application of HPP reduced the initial counts of the examined microorganisms; thus, the microbial counts of mesophilic LAB, lactococci and TVC were found at population levels of 6.45 ± 0.19, 5.83 ± 0.60 and 6.62 ± 0.44 log CFU/g, respectively, at the first batch and 5.45 ± 0.22, 5.83 ± 0.26, 5.30 ± 0.02 log CFU/g, respectively at the second batch, immediately after HPP treatment. Spoilage microbiota (*B. thermosphacta*, *Pseudomonas* sp., *Enterobacteriaceae* and yeast and moulds) was not found at the cheeses since the results from plating were always below <1 log CFU/g (detection limit of the plating method) until the end of storage in both batches examined. In the MEF cheese samples subjected to HPP treatment, the microbiota examined decreased steadily by 0.50 to 1.20 log CFU/g until the end of storage, which depended on the different batches and sampling points. The highest decrease (>1.0 log CFU/g) was observed for lactococci, whilst mesophilic LAB were maintained at higher population levels throughout storage. The differences in the examined microbiota between HPP (control) and HPP-MEF samples were found to be significant for lactococci (*p* < 0.05) at the first batch and for mesophilic LAB and TVC (*p* < 0.05) at the second batch throughout storage.

The pH values recorded until spoilage of the cheese product were found at the expected values (typical for this type of cheese), i.e., 5.70–5.80 (*p* > 0.05) and were not affected by HPP treatment of the cheese slices nor by the addition of MEF (HPP-treated or not) in both batches examined.

### 2.3. Fate of Listeria monocytogenes During Storage

**Figure 2:**
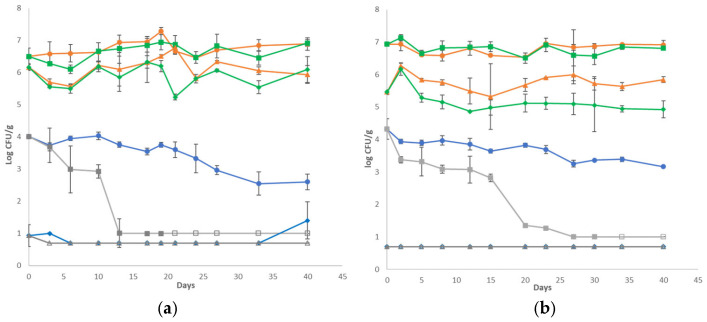
Survival curves of *Listeria monocytogenes* cocktail strains (control: ●, MEF:■, HPP–control: ◆, HPP-MEF: ▲) and TVC (control: ●, MEF: ■, HPP–control: ▲, HPP-MEF: ◆) during storage of cheese slices at 4 °C for batch 1 (**a**) and batch 2 (**b**). Open symbols (□, ◇, △) indicate absence of *L. monocytogenes* after applying the enrichment method. The bars represent the mean values of three cheese replicates ± standard deviations.

Survival curves of *L. monocytogenes* population and growth curves of TVC for every treatment are presented in Figure 2. The initial inoculum level of the pathogen was measured at the cheese slices at 4.01 ± 0.01 and 4.32 ± 0.03 log CFU/g for the two batches, respectively. No growth of *L. monocytogenes* was observed in any treatment throughout storage. The application of HPP resulted in an immediate reduction of approximately 3 log CFU/g compared to samples without HPP treatment. In the first batch, pathogen levels were reduced to 0.94 ± 0.33 log CFU/g, whereas in the second batch, counts were below the detection limit of the plating method (<0.70 log CFU/g) and were confirmed only after enrichment. Linear mixed-effects modelling confirmed a significant treatment × time interaction (*p* < 0.001), indicating distinct decline dynamics among treatments. MEF samples exhibited a progressive reduction over storage, whereas HPP samples remained relatively stable following the initial high reduction after the applied pressure. The combined HPP-MEF treatment did not demonstrate a statistically significant immediate synergistic effect in quantitative counts. In several cases, *Listeria* population levels fell below the detection limit and were detected only after enrichment (open symbols in Figure 2). To appropriately account for these qualitative observations, presence/absence data were analyzed using a binomial generalized linear mixed model. Detection probability was significantly influenced by treatment, storage time, and their interaction (*p* < 0.01), indicating that detection probability decreased differently among treatments over time. Model-predicted probabilities (Figure S2) showed that MEF-treated samples exhibited a gradual reduction in detection probability during storage, whereas HPP-treated samples displayed low and relatively stable probabilities following the initial reduction. The combined HPP+MEF treatment demonstrated a pronounced decrease in detection probability at later storage times, suggesting enhanced long-term suppression compared to single treatments. Mesophilic LAB (*p* > 0.05) and lactococci (*p* > 0.05) populations and pH values were found to be similar to those of the non-inoculated cheese samples throughout storage.

After fitting the survival data of the pathogen using the modified Weibull model, the results indicated a good fit, as is evident in Table 1. Convex (*p* > 1) survival curves of the pathogen were acquired for all cheese cases, indicating a shoulder effect, except for the inoculated control case (only at the second batch), which was close to linear. Regarding the δ rate, the lowest values were obtained for the cheeses supplemented with the MEF, indicating the greater death rate of *L. monocytogenes* in this case. In addition, since the δ parameter indicates the time of the first decimal reduction (10-fold reduction) of the survival population [22], the pathogen had the highest survival rates at the control samples, especially at the second batch, as no decimal reduction was observed during 40 days of storage (δ > 40 days). Inactivation parameters were not determined in inoculated cheeses subjected to HPP since the pathogen was mostly present only after enrichment.

### 2.4. Sensory Evaluation

**Figure 3:**
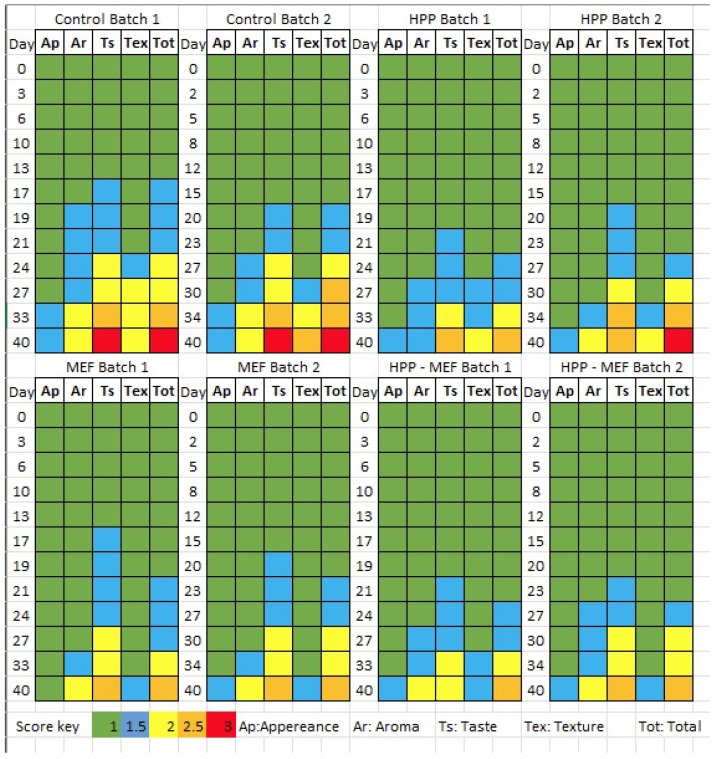
Sensory scores of cheese slices treated or not with HPP, with or without MEF during storage at 4 °C. Appearance (Ap), aroma (Ar), taste (Ts), and texture (Tex) were evaluated using a three-class evaluation scheme with intermediate values (1 = fresh, 1.5 = semi-fresh, 2 = acceptable with clear deviations, 2.5 = spoiled, 3 = unacceptable). Scores represent the mean values of 10 panelists. A score of 1.5 was considered the threshold for the first detectable quality deterioration, while samples were considered unacceptable when at least one attribute reached ≥2 in at least half (5) of the panelists. Total score (Tot) represents the average of the four sensory attributes. Color coding corresponds to the score scale, as indicated in the legend. (The sensory scores of control and HPP samples without MEF have also been included at Papadopoulou et al. [21]).

The findings of the organoleptic evaluation are shown in Figure 3; however, it must be pointed out that the results concerning the scores of the control and HPP samples (without mastic EO, which served as control samples, too) are common with those described in our previous study [21]. Nevertheless, the results related to mastic EO films (MEFs), which constituted the main objective of the present study, are reported here for the first time. The shelf life of cheese slices was estimated as the storage time when the samples retained acceptable sensory properties, while the end of the shelf life was defined as the time point when a sample received total sensory scores > 2. Given that the microbiological changes were limited throughout storage, as described above, the shelf life determination was based primarily on organoleptic evaluation. Overall, the application of HPP lengthened the cheese’s shelf life, as was evident by the higher proportion of fresh/semi-fresh samples in contrast to the other treatments. The presence of MEF films resulted in detectable modifications in the odor and taste of the cheese slices compared to the typical sensory profile of the cheese; however, these changes were generally evaluated positively by the panelists, and this was reflected in a distinct sensory cheese character rather than a uniform increase in total sensory scores. Although all samples exhibited a gradual decline in sensory quality during storage, the HPP-treated samples with MEF maintained higher total scores for a longer period (more fresh/semi-fresh samples). Therefore, the application of HPP improved cheese shelf life and its sensory profile, whereas the incorporation of mastic EO through MEF films provided a product with a unique and positively perceived sensory identity. However, a limitation of the present study is that sensory evaluation was primarily oriented toward shelf life determination rather than detailed sensory profiling of products containing the mastic EO, which could have provided more comprehensive information on its specific sensory attributes in combination with cheese.

### 2.5. Fourier Transform Infrared Analysis

**Figure 4:**
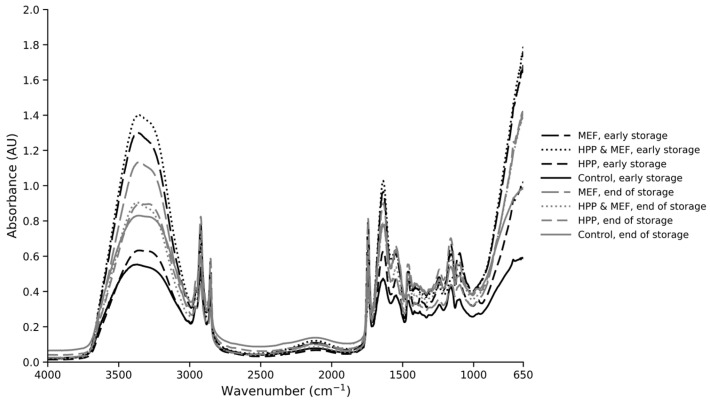
FTIR spectra of 650–4000 cm^−1^ collected from indicative cheese samples (control, MEF, HPP and HPP-MEF) at early and end storage days at 4 °C.

Typical FTIR spectral data collected in the range of 4000–650 cm^−1^ are presented in Figure 4. Figure 4 does not provide special feature peaks that could reflect qualitative differences among the different treatments between samples during storage, and the variations in major components were also limited. As is evident from Figure 4, the major peaks observed in the spectrum were 3340, 2940, 2918, 2854, 1736, 1625, 1530, 1456, 1249, 1159 and 1099 cm^−1^. The 3650–2990 cm^−1^ area (peak at 3340 cm^−1^) is related to proteins (N-H stretch) and hydroxyl groups from water/proteins (O-H stretch), whereas the region between 3000 and 2850 cm^−1^ includes –CH3, >CH2 and >CH-stretching vibrations in fatty acids [23,24]. The area between 1700 and 1500 cm^−1^ is dominated by protein bands, i.e., Amide I; 80% C=O stretch, 10% C-N stretch, 10% N-H bend and H_2_O (O-H) (1640 cm^−1^), Amide II; 40% CN stretch, 60%NH (1550 cm^−1^) and CH_2_ (1456 cm^−1^) bend, while 1750–1700 cm^−1^ is related to C=O stretching vibration of fatty acids esters (1740 cm^−1^) and fatty acids (1711 cm^−1^) [25]. The peak observed at 1249 cm^−1^ (the 1260–1205 cm^−1^ area) includes vibrations in lipids, nucleic acids (asym PO_2_- stretch), and amide III (C-N stretch, N-H bend, and C¼O-N) [26]. The area between 1200 and 900 cm^−1^ is attributed to mono- and polysaccharides due to the C-O stretch bonds related to sugar, where lactose has a distinct peak at 1159 cm^−1^ [24].

Support vector machine models were built to indicate and discriminate differences occurring in the metabolic profiles of the cheese products treated or not with HPP and MEF. These spectral differences were subtle and could only be reliably detected using multivariate analysis. The confusion matrix of the SVM classification model for discrimination among different treatments is shown in Table 2, and satisfactory results were obtained in terms of prediction accuracy (81% and 85%) and sensitivity, i.e., 79% and 90% for control, 94% and 92% for HPP, 76% and 79% for MEF and 73% and 76% HPP & MEF samples, respectively, for WS and PS. As is evident from the results, good discrimination was obtained between control and HPP treatments, rather than between MEF and HPP & MEF samples, where many misclassifications were observed between the latter two classes. In contrast, the performance of the SVM model for the freshness categories illustrated less satisfactory rates (i.e., sensitivity was 98%, 48% and 34% for fresh, semi-fresh and spoiled samples, respectively), and this can be attributed most probably to the imbalance in the training data set. Specifically, the model was trained with a disproportionately high number of fresh samples (over 50% of the dataset) compared to a much smaller number of semi-fresh and spoiled samples. This imbalance limited the model’s ability to accurately predict the latter categories, as it was primarily exposed to fresh samples during training.

The outcome of the validation of the SVM regression (prediction) models between FTIR spectra (WS and PS) and microbial data indicated moderate performance based on the calculated values of PE%, RMSE, B*_f_* and A*_f_* (Table 3). With regard to the model performance for the estimation of the microbial counts, the B*_f_* values were in the range of 1.06–1.11 and were considered acceptable; however, values >1 indicate that the model slightly overestimates microbial counts, which is consistent with the high (over 6 log CFU/g) and stable counts of lactic acid bacteria in cheese throughout storage. The mean deviation between predicted and observed microbial counts (A*_f_* values) were 13% for lactococci, 12% for mesophilic LAB, and 9% for TVC, which limited the accuracy of predictions for small variations, as was shown in the microbiological data, indicating less accuracy between observed and predicted population counts. The predicted accuracy of validation was >75% when using the WS, where the PE% was 67–78% for the PS. A %PE > 70% indicates that the model provides reliable predictions [27] for assessing microbial quality via FTIR spectra, although the moderate RMSE (>0.69) indicates limitations in numerical estimations and a moderate fit of the selected model.

### 2.6. Discussion of Findings 

Cheese production in Greece has a long tradition, with a wide variety of cheese products including both Protected Designation of Origin (PDO) and traditional regional varieties. The product of this study, a light semi-hard cheese produced from a mixture of cow’s milk and goat’s or sheep’s milk, is manufactured using starter cultures, and its microbiota is dominated by LAB and lactococci with a population exceeding 7 log CFU/g, as previously described. Similar findings have also confirmed that semi-hard cheeses demonstrate high proportions of LAB, as shown in studies by Vasileiadou et al. [28] and Pappa et al. [29], which reported mesophilic, LAB, and cocci populations above 7 log after ripening and throughout shelf life.

Essential oils (EOs) are effective antimicrobials for enhancing food safety and eliminating spoilage microbiota, but have limitations in their applications due to their strong aroma, volatility, and potential toxicity at high concentrations [11,30]. Encapsulating EOs in polymer matrices, such as sodium alginate films, can mitigate these issues by preventing direct contact with food. This approach enables the development of edible films and coatings, making biopolymer-based active packaging a promising solution [14]. Based on our results, it was observed that the addition of MEF films lengthened the cheese’s shelf life by eliminating yeast and mould populations and provided distinctive sensory characteristics with intense but acceptable mastic aroma and typical cheese taste. Simsek et al. [31] produced yogurt ice cream by adding mastic gum, and results demonstrated that ice cream containing mastic gum was preferred compared to the control in terms of sensory profile. In contrast, in the work of Mitropoulou et al. [32], where ice cream was produced with the addition of mastic essential oil, most of the products were organoleptically rejected due to bad, intensive and bitter taste. However, in both studies, gum or essential oil was incorporated directly into the food matrix, in contrast with the current study, where the EO was added in the film, allowing a gradual release of the volatile compounds of mastic EO. With regard to the inoculated samples with *L. monocytogenes*, the impact of the antimicrobial activity of MEF was evident since the pathogen population steadily decreased to levels below the detection limit, and accordingly, the pathogen was found sporadically only after enrichment until the end of storage. The Weibull model was used to fit the survival data of the pathogen to describe the fate of *L. monocytogenes*. It was shown that the pathogen had the highest survival rates at the control samples and the lowest survival rates were obtained for the cheeses supplemented with the MEF. In the work of Angelidis et al. [33], the behavior of *L. monocytogenes* strains previously inoculated on a grated cheese in different inoculation levels and storage temperatures was studied. Results showed that despite no growth being observed, the pathogen was detectable after nine months of post-inoculation in cold storage. The Weibull model provided a good fit for describing the inactivation kinetics of *L. monocytogenes* across the various experimental conditions examined in this study [33]. However, in their study, the survival of *L. monocytogenes* in grated cheese was examined without the application of inactivation treatments, and predictive microbiology was used as a tool to describe microbial inactivation on cheeses during long storage, in contrast with our study, where different inactivation treatments (HPP, EO) were implemented.

The antimicrobial activity of a variety of EOs and encapsulated systems in cheeses against foodborne pathogens has been studied in previous research. However, limited, if any, research is available for mastic EO in dairy products. Regarding other EOs, the work by Seydim et al. [34] examined the antimicrobial impact of oregano and garlic EOs (2%) after incorporation in whey protein edible films against several foodborne pathogens during 15 days of cold storage of sliced kashar cheese. It was shown that a reduction of 1.19 log CFU/g was evident after 24 h of storage with edible films containing 2% essential oil, while after 15 days of storage, the pathogen was reduced by ca. 2 log CFU/g, the reduction was greater compared to the control samples, and garlic EO was more effective than oregano EO. Polat Yemiş et al. [35] explored the effect of sodium alginate edible coatings with myrtle EO against *L. monocytogenes* during 24 days of cold storage of fresh kashar cheese and noticed a 0.4 and 0.8 log CFU/g reduction in *L. monocytogenes* population throughout storage of cheese coated with 1% and 2% myrtle essential oil, respectively. Those results are in accordance with the findings of this study, where the antimicrobial activity of mastic EO suppressed pathogen growth by ca. 4 log CFU/g after 19 days of storage. Dannenberg et al. [14] used cellulose acetate films with encapsulated pink pepper EO in various percentages (2, 4, and 6%) and investigated their antimicrobial effect on sliced mozzarella cheese inoculated with several foodborne pathogens (4 log CFU/g initial inoculum level) during 12 days of cold storage. Based on their results, the pathogen population significantly decreased in cheese slices at concentrations of 4 and 6% of EO, in contrast to the concentration of 2%, where *L. monocytogenes* decreased by 1 log CFU/g until day 3 and then exhibited a slight increase of 0.5 log CFU/g until the end of storage. In many of these studies, an inhibitory effect on a variety of foodborne pathogens was recorded, highlighting the antimicrobial effect of different EOs and their potential after inclusion in active packaging. The findings of this study are in line with previous studies, where the antimicrobial activity of mastic EO (without HPP) suppressed pathogen growth by ca. 4 log CFU/g until the end of the 40-day storage period. Nevertheless, the EO alone cannot eliminate pathogens, so adding hurdles to promote synergistic effects can be a better option. In this sense, the application of HPP can be a suitable solution.

HPP is a non-thermal emerging technology that has been widely studied to extend the shelf life of perishable food commodities, such as cheese products. However, most of the scientific articles have examined fresh cheeses or cheeses produced using unpasteurized (raw) milk. For instance, Inácio et al. [36] studied various combinations of HPP treatment and time on the microbiological and physicochemical quality of cheeses artificially inoculated with *Listeria innocua* (initial inoculum level of 8.5 log CFU/g) made from raw milk during storage (100 days at 4 °C). They observed that pressure levels of 600 MPa reduced the spoilage and pathogenic microbiota (i.e., *L. innocua*, *Enterobacteriaceae*, yeasts and moulds) under the detection threshold of the plating method, whereas LAB showed a small decline of 0.8 log CFU/g immediately after HPP application and observed a further decrease during storage [36]. Evert-Arriagada et al. [37] examined the inactivation of artificially inoculated (3–4 or 6–7 log CFU/g in cheese) *Listeria* spp. (*L. innocua*, *L. monocytogenes* CECT 4031 and *L. monocytogenes* Scott A) on a starter-free fresh cheese after HPP (300, 400, 500 and 600 MPa, 5 min, 6 °C) and during cold storage. Based on their results, higher pathogen inactivation was evident when higher pressure levels were applied; however, the most pressure-resistant strain was *L. monocytogenes* Scott A. In the same study, it was shown that during cold storage, the counts of *L. innocua* and *L. monocytogenes* CECT 4031 gradually increased during storage to reach high population levels, in contrast to the population of *L. monocytogenes* Scott A that was maintained near the estimated population levels after the HPP [37]. In addition, Tomasula et al. [10] reported the effectiveness of HPP treatment (600 MPa, 20 min, 20 °C) to eliminate *L. monocytogenes* (cocktail of five strains) inoculated on the surface or in the curds of Queso Fresco slices. They observed that HPP was effective for the immediate pathogen reduction to below the detection limit, though recovery of the injured cells and consequently an increase in the pathogen population was observed after 1 and 4 weeks, respectively, for the surface and the curds of cheese slices [10]. In the current study, HPP reduced *L. monocytogenes* population by 3 log CFU/g at the first batch and below the detection threshold of the plating method at the second batch; however, injured cells were able to repair and thus be viable in low population numbers sporadically during storage. Thus, it is evident that while HPP resulted in high inactivation of *Listeria*, as reported in the aforementioned studies and also in the current study, the pathogen can still recover from injury and proliferate during cold storage. In that case, an additional hurdle may be required, i.e., active packaging (AP) incorporated with natural antimicrobial compounds for a synergistic antimicrobial effect combining HPP and AP. In this sense, Gonçalves et al. [38] studied the effectiveness of combining HPP and AP with oregano EO on Coalho cheese experimentally contaminated with several foodborne pathogens, including *L. monocytogenes,* and stored under refrigeration for 21 days. Their results verified that the combination of AP and HPP (400 MPa, 10 min) obtained a greater elimination in the pathogen’s population immediately after processing and throughout cheese storage, where the individual use of AP or HPP allowed bacterial multiplication during storage [38]. Similarly, in the present study, HPP caused considerable reductions of up to 4 log CFU/g (depending on the batch) in the *Listeria* population at time zero, while the combination with MEF kept the counts below the detection threshold of the plating method throughout storage in both batches examined. Based on the results, the applied hurdle combination (MEF combined with mild HPP) effectively inhibited pathogen proliferation and prevented population recovery throughout storage. However, *L. monocytogenes* was occasionally detected after enrichment, indicating survival at very low levels rather than complete eradication. According to the amended EU Regulation (EU) 2024/2895 of Regulation (EC) No. 2073/2005, enrichment detection is considered as presence for regulatory purposes. Therefore, sporadic enrichment-positive results would not comply with the default criterion of “not detected in 25 g” throughout shelf life. Nevertheless, quantitative results demonstrated that pathogen levels remained consistently below 100 CFU/g and no growth was observed during storage. These findings suggest that the combined treatment effectively controls pathogen proliferation. For industrial application, additional validation (e.g., challenge testing, predictive modelling, or extensive shelf life studies) would be required to demonstrate compliance with the applicable microbiological criteria under real production conditions.

Considering that the organoleptic characteristics of the cheese products can vary after the HPP treatment and/or the addition of the associated EO, a sensory assessment of those products is crucial. The EOs have an intense aroma and may alter the typical profile of the food that they are added to, so a threshold of sensorial acceptability by the consumers exists. The results of the current work highlighted the differentiated sensory properties of the cheeses packaged with MEF, where the HPP-treated samples packaged with MEF had the best organoleptic profile. The inclusion of the EO in edible films masked the intensity of its flavor, whereas HPP did not have a negative impact on the cheese slices’ appearance. Limited studies have explored the differentiation in sensory properties after the sole application of edible films or coatings incorporated with EOs, where the combination of HPP and AP is even more rarely examined. Polat Yemiş et al. [35] reported that coating Kashar cheese with myrtle EO (2%) had moderate acceptability due to the intense smell and taste of the EO, in contrast to the current work, where the concentration of 2% of EO in the edible films was acceptable. In addition, Cano Embuena et al. [39] assessed the sensorial properties of a coated goat cheese with chitosan–oregano EO films, and the results revealed that the double-coated cheeses were graded with the highest organoleptic scores. Bleoancă et al. [6] evaluated the appropriate concentration of thyme extracts applied to fresh cheese by a consumer’s sensorial study and observed that the concentration acceptable for cheese was lower than the concentration required to eliminate *L. monocytogenes;* however, the cheese was not evaluated after the application of HPP and thyme extract.

The use of rapid non-destructive techniques, such as spectroscopic techniques, has been implemented to overcome the limitations of conventional food microbiology and automatically monitor the food processes in all stages [16]. FTIR is a promising analytical technique employed in the rapid and quantitative monitoring of food safety and quality, and when combined with appropriate computational methods, it can provide rapid estimations related to food spoilage and safety. With regard to cheese products, the AOAC International [40] has validated FTIR as a method for determining the protein, lactose, fat, and total solid contents in specific dairy products, and since then, several studies have evaluated cheese quality in terms of ripening, composition, and authenticity using FTIR. FTIR combined with multivariate statistical tools has been successfully implemented in monitoring butter cheeses’ authenticity [41], in assessing the sensory qualities of Norwegian cheeses during storage [42] and Cheddar cheese quality and composition [43], in estimating the microbiological quality and sensory attributes during feta cheese storage [18], in discriminating grated Parmigiano Reggiano cheeses from other grana-type cheeses [23], or in discriminating cheeses based on their ripening time [44,45,46]. Among those works, Kraggerud et al. [42] had unsatisfactory predictions of sensory traits, probably due to the subjective evaluation of the sensory attributes, which are not directly associated with a specific infrared region, a result that was also apparent in the present work. On the contrary, Subramanian et al. [43], who studied the composition and organoleptic quality of numerous Cheddar cheese samples obtained from local stores, observed that the regression models (partial least squares regression) revealed a good fit and accurate determination of their physicochemical contents, as well as that the samples were very well categorized based on their sensory quality. However, none of the above works estimated the microbiological characteristics of cheeses during storage, except for the previous work in Feta cheese, where PLS-R models were successfully employed to predict the counts of the examined microbiota present throughout Feta cheese storage [18]. In the current work, a different approach was followed using support vector machine analysis, and the obtained results were acceptable for microbial estimation and the discrimination between different treatments but less satisfactory for the sensory discrimination according to the performance indices described above, which may be considered a limitation of the findings of the present study. The latter performance indices values, however, do not overrule FTIR’s potential as a reliable source for cheese quality control. Indeed, FTIR data have shown great potential in the assessment of feta cheese microbiological quality [18] or in predicting the sensory quality of Norwegian cheeses [42]. Nevertheless, additional research is necessary to make this approach suitable in cases regarding novel packaging and/or different treatment methods that are increasingly being applied, which consequently alter both the food matrix and the nature of spoilage. However, no other potential comparisons with relevant studies could be made, since most of the studies monitored biochemical changes throughout cheese ripening, while in the current study, the changes in the spectra were monitored throughout storage and after HPP treatment.

## 3. Conclusions

The mastic EO exhibited antimicrobial activity in vitro against certain Gram-positive bacteria and yeast strains after incorporation in sodium alginate edible films applied to cheese slices. Hence, the presence of the antimicrobial edible film was crucial in reducing spoilage microbiota during storage. Organoleptic assessment showed that the panel evaluated the cheese combined with the mastic EO positively, especially the samples pretreated with HPP, highlighting their potential for industrial application. In experimentally contaminated sliced cheese with *L. monocytogenes*, the combination of HPP and MEF was the most effective approach for controlling pathogen growth. Although complete eradication was not always achieved and sporadic detection after enrichment indicated survival at very low levels, the applied hurdle strategy effectively inhibited pathogen proliferation under the tested conditions. These findings support the use of mild combined processing and natural antimicrobials as a strategy to contribute to improved control of *L. monocytogenes* in RTE cheese, while preserving product quality. Finally, FTIR spectroscopy coupled with data analytics enabled the discrimination of metabolic and structural differences among treated and untreated cheese samples, which were not evident by visible inspection of the spectra. The developed model demonstrated predictive potential for microbiological counts, indicating its applicability to serve as a practical tool for the dairy industry to monitor product quality. Moreover, the model was able to highlight the differences between the treatments caused by the induced modifications of the cheese matrix, providing additional insight into how the applied hurdles can change the food matrix and the character of the spoilage. Overall, the proposed multi-hurdle approach offers a practical and innovative framework for improving safety, sensory quality, and shelf life of cheese, while reducing processing intensity.

## 4. Materials and Methods

### 4.1. Bacterial and Yeast Strains

In the present study, a variety of strains (typical for cheeses), including spoilage-specific organisms, common pathogenic bacteria, and yeasts, were examined (Supplementary Table S1). The 17 strains were the following: *Enterococcus faecium* SMX4, *Leuconostoc mesenteroides* FMX3, *Lactobacillus plantarum* T75 (collection of ITAP, HAO—DIMITRA), *Listeria monocytogenes* FMCC-B-128, FMCC-B-129, FMCC-B-133 (FMCC strains—collection of the laboratory of Microbiology and Biotechnology of Foods, Agricultural University of Athens, Greece), *Listeria monocytogenes* DSM15675, DSM19094 (The Leibniz Institute DSMZ—German Collection of Microorganisms and Cell Cultures GmbH, Braunschweig, Germany), and the yeasts *Pichia fermentans* OZ1, OZ3, KZ5, KZ10, KZ11, KZ12, *Candida zeylanoides* KZ7, KZ8, and *Yarrowia lipolytica* OZ5 (previously isolated from spoiled cheeses, collection of ITAP, HAO-DIMITRA).

The bacteria and yeasts were grown in the appropriate media and conditions relevant for each species. Pathogens were incubated in Brain Heart Infusion Broth (LAB049, LabM, Lancashire, UK) at 37 °C for 18 h, lactic acid bacteria (LAB) strains were incubated in MRS broth (4017292, Biolife, Milano, Italy) at 30 °C for 24 h and yeasts were incubated in Potato Dextrose Broth (NCM0157A, Neogen Corporation, Lansing, MI, USA) at 25 °C for 48 h. The cells were then centrifuged, the pellet was washed using ¼ strength Ringer’s solution (Ringer solution Tablets, 96724- 100TAB, Merck, Darmstadt, Germany) and finally a Ringer’s solution containing each of the examined microorganisms was prepared at a final population level of 5 log CFU/mL.

### 4.2. Antibacterial Assays and Measurement of the Minimum Inhibitory Concentration by Optical Density Method

The 17 selected strains (Supplementary Table S1) were evaluated for their survival in 22 concentrations varying from 0.006% to 2% (*v*/*v*) of mastic EO, and the antimicrobial activity of the EO was examined in vitro using the optical density method according to Chorianopoulos et al. [47]. In short, the growth of the strains was recorded by means of changes in the optical density of the strains’ suspensions supplemented with mastic EO in multiple concentrations, as described below. Four stock solutions of (2.0, 1.8, 1.5 and 0.3% *v*/*v*) were prepared by adding the appropriate quantity of EO and the appropriate broth for each strain (as described above) and mixing well using a homogenizer (WiseTis HG-15A SET B, WITEG, Wertheim, Germany) to make a homogenous solution (4000 rpm, 2 min). Immediately after mixing, aliquots (180 μL) were added to the wells of a 96-well microplate, and half-fold serial dilutions followed using the same broth medium to cover the range from 2% to 0.006% *v*/*v* mastic EO. Each strain was added to the wells (prefilled with the appropriate culture medium containing the mastic EO) in a final population of 3 log CFU/mL. The microplates were incubated for up to 72 h, at 25, 30, or 37 °C (depending on the tested strain), and OD measurements were taken at 610 nm in a microplate reader (SpectraMax Plus 384 Microplate Reader, Molecular Devices, Sunnyvale, VA, USA). Each microorganism was tested three times for two independent replicates for each of the 22 EO concentrations. Values of the Minimum Inhibitory Concentration (MIC) were estimated using the Lambert and Lambert model [20], as described in detail by Chorianopoulos et al. [47].

### 4.3. Experimental Design and Preparation of the Non-Inoculated and Inoculated Cheese Slices

Cheese slices (low fat) weighting 25 g (dimensions of 10 cm × 8 cm) were obtained from a supermarket (Athens, Greece) and transported immediately to the laboratory and consequently treated based on the eight different treatments (Table 4) examined in the present study. Two independent experiments (two batches, seasonal variations) with triplicate samples (276 samples for each batch, 552 samples in total) were performed. All samples were divided equally in all cases examined.

The cases examined in the current study were the following: cases involving the employment of sodium alginate edible films enriched with mastic EO (Mastic Edible Films (MEFs), described below) with or without the addition of *L. monocytogenes*. Slices of cheese without MEF and without the pathogen were prepared to serve as control cases and were presented in our recent study [21]. Cheese slices (with or without the pathogen) were subjected to HPP treatment (500 MPa, 2 min) and then samples were divided into those packed with MEF or without (to serve as control samples).

A three-strain cocktail of *L. monocytogenes* (based on the results acquired by the in vitro assay, Section 2.1) was used to inoculate the cheese slices (at both sides of the slice) at a final population level of ca. 4 log CFU/g, before any treatment. In brief, monocultures of the strains FMCC-B-133, DSM15675 and DSM19094 were recovered from a stock culture (−80 °C) and incubated at 37 °C for 24 h in BHI (LabM) broth; accordingly, the rejuvenated culture was incubated for 18 h under the same conditions (selection of the strains was based on their different ecological origin, i.e., FMCC B133 is a foodborne isolate from soft cheese, DSM 15675 is a clinical isolate and DSM 19094 is an isolate coming from food processing environment). Each strain was centrifuged, the pellet obtained was washed using ¼ strength Ringer’s solution (Merck), and finally, 10 mL of Ringer’s solution containing the three strains was prepared. This final solution was prepared by mixing, in equal volumes, the three monocultures of the strains and was used to inoculate slices of cheese. Each slice of cheese was inoculated with 4 log CFU/g (0.01 mL of a 10^6^ dilution of the cocktail strain culture and then spread on both cheese sides of each slice). The inoculum level was defined by spreading the same dilutions on Palcam Agar (BK145HA, Biokar Diagnostics, Allonne, France) and Petri dishes were incubated at appropriate conditions (37 °C for 48 h). Cheese slices were also prepared without the addition of the pathogen to serve as another control. One storage temperature (4 °C) was examined, while cheese samples were vacuum packed into polyamide plastic pouches (thickness 90 μm, gas permeability [cm^3^/m^2^. day·bar at 20 °C, 50% RH] was ca. 90, 25, and 6 for CO_2_, O_2_, and N_2_, respectively; Flexo-Pack S.A., Athens, Greece), using a HenkoVac 1700 Machine (Howden Food Equipment B.V., ‘s-Hertogenbosch, The Netherlands).

### 4.4. High-Pressure Processing (HPP) Treatment

The HPP conditions employed at half of the samples with and without the pathogen were 500 MPa for 2 min at room temperature (20–22 °C), and those parameters were selected based on the literature findings [48] and after preliminary experiments to evaluate the effectiveness of different HPP treatments. In that sense, preliminary inactivation experiments were conducted using pressure levels of 400 MPa for 2 and 5 min, 500 MPa for 2 and 5 min and 600 MPa for 2 and 5 min and microbiological analysis (indigenous microbiota and *Listeria* inactivation) followed at days 0, 3, 8 and 15 of storage. Based on these results and focusing primarily on maintaining the sensorial integrity of the product and ensuring that cheese slices could be easily separated after HPP treatment, the main experiment was then designed, and the selected HPP conditions were defined. HPP treatments were carried out using a Food Pressure Unit (FPU 1.01, Resato International BV, Roden, The Netherlands), consisting of a 250 mL central vessel with a maximum operating pressure of 1000 MPa. Samples were vacuum-packaged and processed immersed in the pressure-transmitting fluid (polyglycol, ISO viscosity class VG 15; Resato International BV). The pressure come-up rate was approximately 20 MPa/s (≈100 MPa per 7 s), and pressure release occurred within approximately 3 s. The reported holding time (2 min) corresponds exclusively to the period during which the target pressure (500 MPa) was maintained and does not include the come-up or decompression time. Pressure and temperature were continuously monitored and recorded at 1 s intervals throughout the process. The inoculated cheese samples were stored at 4 °C for approximately 1 h to allow bacterial attachment and were pre-cooled before pressurization to minimize the temperature rise due to adiabatic heating. Based on system specifications and preliminary measurements, adiabatic heating during compression at 500 MPa resulted in an estimated temperature increase of approximately 10–12 °C. Additional technical details of the HPP system have been previously described by Argyri et al. [49].

### 4.5. Edible Films Preparation and Application in the Cheese Slices

The sodium alginate edible films were prepared according to Pavli et al. [13]. In brief, 2% of sodium alginate (A3249, AppliChem GmbH, Darmstadt City, Germany) solution was prepared with distilled water, and 1% of glycerol was used as a plasticizer. Mastic EO in 2% *v*/*v* (donation of Chios Mastic Gum Growers Association L.L.C., Chios, Greece) was dispersed to the forming solution by high speed homogenization (26 × 10^3^ rpm for 3 min) (Ultra Turrax; IKA Staufen, Staufen im Breisgau, Germany) and the films (Mastic Edible Films (MEFs)) were produced by adding 20 g of the forming solution in square Petri dishes. The films were dried overnight (20–22 °C, ambient temperature) inside a laminar flow cabinet. After the drying process, the films were detached from the dishes by adding 1 min calcium chloride (A1873, AppliChem) solution (2% *w*/*v*), dried to remove excess water, and immediately applied to both sides of the cheese slices.

On the cheese slices that were not treated with HPP, MEFs were used to cover both sides of the cheese slices, and then the cheeses were packaged under vacuum and cold stored (4 °C). For the HPP-treated samples, cheese slices were covered on both sides with the MEF after the HPP processing and then vacuum-packed and cold stored. The same treatment was followed for the inoculated cheese samples with the pathogen.

### 4.6. Microbiological Analyses and pH Determination

Microbial analysis and pH determination of all cheese cases were performed at twelve (12) specific sampling points throughout storage at 4 °C for up to 40 days, as described in detail by Papadopoulou et al. [21]. The intervals between samplings ranged from 2 to 6 days and were adjusted according to the progression of cheese spoilage. In brief, 10 g portions of triplicated cheese samples (from each batch) with 90 mL of sterilized ¼ Ringer’s solution were homogenized for 60 sec at room temperature in a stomacher (Stomacher 400 circulator, Seward Limited, Norfolk, UK), resulting in an initial dilution of 10^−1^. For the HPP-treated samples and the MEF samples, a reduced initial dilution, i.e., 10 g of cheese and 50 mL of Ringer’s solution, was applied, and samples were homogenized accordingly. This procedure was used to improve the sensitivity of the enumeration method and to lower the detection limit to approximately 0.7 log CFU/g. The resulting suspensions were subjected to serial dilutions in the Ringer’s solution and 0.1 mL or 1 mL of the samples were spread or poured in triplicate on Plate Count Agar (CM0325, Oxoid, Basingstoke, UK) for the enumeration of Total Viable Aerobic Counts (TVCs), MRS ISO Agar (NCM0190A, Neogen, Lansing, MI, USA) for the enumeration of mesophilic LAB, M17 Agar (4017192, Biolife Italiana, Milano, Italy) for the enumeration of lactococci, Pseudomonas Agar Base (CM559, Oxoid) supplemented with CFC selective supplement (SR0103, Oxoid) for the enumeration of *Pseudomonas* spp., Streptomycin Thallous Acetate Actidione Agar (4020792, Biolife) with STAA selective supplement (4240052, Biolife) for the enumeration of *Brochothrix thermosphacta*, Violet Red Bile Glucose Agar (CM 0485, Oxoid) for the enumeration of *Enterobacteriaceae*, Rose Bengal Chloramphenicol Agar (BK151HA, Biokar Diagnostics, Allonne, France) for the enumeration of yeasts and moulds, and Palcam Agar Base (NCM0111A, Neogen) with selective supplement (NCM4041-0.5 Palcam PAC Supplement, Neogen) for the enumeration of *Listeria monocytogenes*. When *L. monocytogenes* counts were found below 1.0 log CFU/g in the inoculated samples (regardless of the HPP treatment), enrichment according to ISO 112901:2017 [50] was applied to ensure its presence/absence. This threshold was selected to ensure sensitive detection of low population presence. For HPP (without or with the addition of MEF)-treated samples, enrichment was applied from the first sampling point, whereas for MEF samples, enrichment was applied from the 13th day and onwards. In the HPP-treated cheese samples, the incubation time in all the examined growth media was extended by 1–2 days to allow the recovery of the injured or stressed cells by HPP treatment, according to Argyri et al. [51]. On the non-inoculated cheese samples, enrichment methods were applied to detect the presence/absence of *Salmonella* sp. according to ISO 6579-1:2017 [52] and *L. monocytogenes* according to ISO 112901:2017.

### 4.7. Estimation of L. monocytogenes Inactivation Kinetic Parameters

The modified Weibull model (GInaFiT Excel add-in version 1.6 implemented in Microsoft Excel) was applied using GInaFiT software version 1.6 [53] according to Albert and Mafart [22] to describe the survival kinetics of *L. monocytogenes* in sliced cheese during refrigerated storage.

The model was fitted to the log reductions of *L. monocytogenes* over the 40-day storage period under different treatments. Goodness-of-fit was evaluated using the root mean square error (RMSE) and the adjusted coefficient of determination (R^2^adj). Parameter values (δ: scale parameter and *p*: shape parameter) were estimated for each treatment condition to characterize the survival kinetics.

### 4.8. Sensory Assessment

The organoleptic assessment of the cheese samples without the pathogen was carried out by a semi-trained panel consisting of 10 people (staff from ITAP laboratory), at the same point as the microbial analyses, as already described by Papadopoulou et al. [21]. All panelists provided their consent before participating in the study. Prior to evaluation, MEFs were removed from the cheese slices. The selected criteria for organoleptic assessment were appearance (color), aroma, and taste. A three-class evaluation scheme was used to assess the organoleptic properties: the first category (0–1, fresh) indicated no off-flavors and a typical cheese appearance; the second category (1.5–2, semi-fresh) reflected a mild acidic taste and smell, though the quality was still deemed acceptable; and the third category (2.5–3, spoiled) denoted an intense acidic aroma and taste, indicating the product was no longer acceptable (sensory scores represent the average of 10 panelists’ ratings). A score of 1.5 was considered the threshold where the first clear deviations from freshness were perceived, including mild acidification in taste or odor, though the product was still acceptable. Samples were deemed unacceptable if at least half of the sensory panel (5 persons) rated any single attribute negatively (≥2). This time point was identified as the end of the sample’s shelf life. Control samples (commercial cheese) were included in all sensory sessions.

### 4.9. FTIR-ATR Analysis

A PerkinElmer Frontier FTIR Spectrometer (PerkinElmer, Inc., Waltham, MA, USA) was used for the analysis of the cheese samples after the end of microbiological analysis (after removal of the MEF). FTIR spectra of the surface of cheese samples were collected using a ZnSe 45° attenuated total reflectance (ATR) flat plate crystal with a Horizontal ATR (HATR) sampling accessory at room temperature, over the wavenumber range 4000 to 650 cm^−1^. To achieve the best possible contact with the crystal’s surface, the tested cheese samples were transferred to the crystal plate and then pushed using the machine’s gripper. Scans per measurement were ten (10), with a resolution of 4 cm^−1^. Reference spectra were acquired by collecting a background spectrum from the cleaned blank flat plate crystal (ZnSe 45°) before each measurement of the sample. A total of 546 FTIR spectra (samples without the pathogen, 3 replicates per cheese sample for each case, i.e., cheese with no treatments, MEF, HPP-treated and a combination of HPP and MEF) were collected. FTIR spectra of 4000 to 650 cm^−1^ (whole spectra, WS) and 1800 to 900 cm^−1^ (Partial spectra, PS) were further employed in multivariate analysis.

### 4.10. Data Analysis and Evaluation of Model Performance

Data exploration and interpretation were based on support vector machine (SVM) analysis, using TIBCO Statistica, version 14 (TIBCO Software Inc. 2020. Data Science Workbench, Palo Alto, CA, USA). The standard normal variate transformation was used prior to data analysis, for the background correction of the FTIR spectral data in the two selected regions (WS: 4000 to 650 cm^−1^ and PS: 1800 to 900 cm^−1^). For the training of the model, the first batch (N = 269) was employed, while for the external validation of the model, the second batch (N = 277) was used. For the SVM classification, the normalized FTIR spectral data of the selected regions (WS and PS) were used as an input in the SVM classification model, and the outputs were (i) the different treatments (control, HPP, MEF, HPP-MEF) and (ii) the predefined three sensory classes (F, SF, S). The acquired results were used to build confusion matrices and further evaluate the model’s performance by calculating sensitivity and overall correct classification (accuracy %).

SVM regression (SVM-R) models were developed to correlate the microbial counts (TVC, mesophilic LAB and lactococci) with the FTIR data. The latter were used as input variables to train the SVM-R, and the output was the microbial population (in log CFU/g) of each of the selected microbial groups. The selection of SVM regression and classification types and parameter optimization of the models were performed according to Papadopoulou et al. [19]. The performance indices used for the efficacy of the regression model (SVM-R) were the root mean square error (RMSE), the bias (B*_f_*) and accuracy (A*_f_*) factors and the accuracy of prediction (PE%, the percentage of samples correctly predicted). To determine whether individual prediction errors were acceptable, the prediction zone (−1.0 log< acceptable PE < 0.5 log) was calculated, as described in detail in a study by Papadopoulou et al. [18].

### 4.11. Statistical Analysis

Microbiological data and pH values were expressed as means ± SD of the three replicates of the two batches. Univariate analysis (one-way analysis of variance (ANOVA)) and post hoc analysis-Tukey’s test were employed to compare mean values of microbiological and pH data and determine significance using StataMP 17 (StataCorp., LLC, College Station, TX, USA). Statistical analyses were performed in R (R version 4.5.1; R Foundation for Statistical Computing, Vienna, Austria). Quantitative *L. monocytogenes* counts (log CFU/g) were analyzed using linear mixed-effects models (LMMs) implemented in the *lme4* and *lmerTest* packages. Treatment, storage time (treated as a continuous variable), and their interaction were included as fixed effects, while batch was included as a random effect to account for variability between independent productions. Presence/absence data, including samples detected only after enrichment, were analyzed separately using generalized linear mixed models (GLMMs) with binomial distribution and logit link functions. Treatment, storage time, and their interaction were included as fixed effects, and batch was included as a random effect. Storage time was treated as a continuous variable to account for the unbalanced sampling schedule between batches and to model differences in decline dynamics among treatments. Samples below the enumeration detection limit were not replaced with arbitrary values for parametric analysis. Instead, qualitative detection data (including enrichment-only positives) were incorporated in the binomial GLMM framework, thereby avoiding bias associated with substitution-based approaches for censored data. Estimated marginal means and pairwise comparisons were obtained using the *emmeans* package, with Tukey adjustment for multiple comparisons where appropriate. Statistical significance was set at *p* < 0.05.

## Figures and Tables

**Table 1:** Parameter estimation (mean ± standard error) of the modified Weibull model used for fitting the survival of *Listeria monocytogenes* on cheese slices packaged with or without edible films supplemented with Chios mastic gum essential oil at 4 °C.

Cheese Cases Inoculated with *L. monocytogenes*	N_0_ ^a^ (log CFU/g)	N_res_ ^b^	*δ* (Days) ^c^	*p* ^d^	R^2^ adj ^e^
Control, batch 1	3.90 ± 0.06	2.57 ± 0.10	26.47 ± 1.08	14.22 ± 1.26	0.940
Control, batch 2	4.21 ± 0.14	na ^f^	40.05± 0.01	0.73 ± 0.55	0.806
MEF, batch 1	3.62 ± 0.16	0.99 ± 0.10	10.41 ± 0.67	5.18 ± 1.96	0.957
MEF, batch 2	3.29 ± 0.04	1.00 ± 0.03	17.98 ± 0.35	3.56 ± 0.33	0.996

^a^ Initial population. ^b^ Residual population. ^c^ Scale parameter. ^d^ Shape parameter. ^e^ Adjusted coefficient of determination. ^f^ Not applicable.

**Table 2:** Confusion matrix of support vector machines classification models regarding treatment discrimination of cheese samples based on FTIR spectral data (650–4000 cm^−1^ [WS] and 900–1800 cm^−1^ [PS]).

From/To	Control	HPP	MEF	HPP & MEF	Total	Sensitivity (%)
WS						
Control	57	10	4	1	72	79.17
HPP	4	68	0	0	72	94.44
MEF	0	0	50	16	66	75.76
HPP & MEF	0	0	18	49	67	73.13
		Correct classification (accuracy, %)				80.87
PS						
Control	65	3	3	1	72	90.27
HPP	6	66	0	0	72	91.66
MEF	1	0	52	13	66	78.78
HPP & MEF	0	0	16	51	67	76.11
		Correct classification (accuracy, %)				84.83

**Table 3:** Calculated performance indices for prediction of TVC in cheese samples using predicted estimates of the external validation dataset from support vector machine regression (SVM-R) models based on FTIR spectral data (650–4000 cm^−1^ [WS] and 900–1800 cm^−1^ [PS]).

Spectra	Microbial Group	B*f*	A*f*	RMSE	Accuracy of Prediction (%)
WS	TVC	1.06	1.08	0.69	83.03
	mesophilic LAB	1.09	1.12	0.88	75.09
	lactococci	1.06	1.13	0.89	75.81
PS	TVC	1.08	1.09	0.75	78.70
	mesophilic LAB	1.11	1.13	0.95	72.92
	lactococci	1.10	1.13	0.95	67.15

**Table 4:** Experimental design indicating the eight treatments applied to cheese slices.

Cases	Control (Cheese)	HPP	MEF	*L. monocytogenes*
1	√			
2		√		
3		√	√	
4			√	
5	√			√
6		√		√
7			√	√
8		√	√	√

All treatments were tested in two independent batches with triplicate samples.

## Data Availability

The dataset is available upon request from the authors. The authors have reviewed and edited the output and take full responsibility for the content of this publication.

## Supplementary Materials

The following supporting information can be downloaded at: https://www.mdpi.com/article/10.3390/gels12030255/s1, Table S1: List of examined microorganisms in vitro; Table S2: MIC values and their standard errors from optical density technique (% *v*/*v* concentration of Chios mastic gum essential oil in broths using 3 log CFU/mL inoculum level of each microorganism); Figure S1: The inhibition profile of Chios mastic gum essential oil against *Listeria monocytogenes* FMCC-B-133 with optical density technique. ● Dots represented mean values of 6 replicates; Figure S2: Model-predicted probability of *Listeria monocytogenes* detection during storage of cheese slices at 4 °C. Probabilities were estimated using a generalized linear mixed model (binomial distribution, logit link), including treatment, storage time, and their interaction as fixed effects and batch as a random factor. Shaded areas represent 95% confidence intervals. Detection probability incorporates both direct enumeration and enrichment-only positive samples.

## Abbreviations

The following abbreviations are used in this manuscript:

HPP	High-Pressure Processing
FTIR	Fourier Transform Infrared spectroscopy
MEF	Mastic edible films
MIC	Minimum Inhibitory Concentration
SVM	Support Vector Machines
EO	Essential Oil
AP	Active Packaging
PS	Partial spectra
WS	Whole spectra
