# Supplementary material for: Chios Mastic Essential Oil in Sodium Alginate Edible Films Combined with High-Pressure Processing as Listeria monocytogenes Inhibitors in Cheese Slices
Source: Gels. 2026 Mar 18;12(3):255. doi: 10.3390/gels12030255 (PMC13025587; doi:10.3390/gels12030255)
Supplement: Supplementary file 1 [file gels-12-00255-s001.zip › gels-4154965-supplementary.pdf]

Supplementary Figures and Tables

Supplementary Table S1: List of examined microorganisms *in vitro* .

**Microorganism**	**Isolation source**	**Reference**
Listeria monocytogenes DSMZ15675	Soft cheese	The Leibniz Institute DSMZ - German Collection of Microorganisms and Cell Cultures GmbH
Listeria monocytogenes DSMZ19094	Poultry	The Leibniz Institute DSMZ - German Collection of Microorganisms and Cell Cultures GmbH
Listeria monocytogenes FMCC-B-128	Soft cheese	Food Microbiology Culture Collection (FMCC), Agricultural University of Athens (AUA)
Listeria monocytogenes FMCC-B-129	RTE frozen meat meal	FMCC, AUA
Listeria monocytogenes FMCC-B-133	Soft cheese	FMCC, AUA
Lactiplantibacillus plantarum T75	Xerotiri cheese	Pavli et al., 2016
Leuconostoc mesenteroides FMX3	Feta PDO cheese	Kamarinou et al., 2022
Enterococus faecium SMX4	Semi hard cheese	Kamarinou et al., 2022
Pichia fermentans OZ1	Feta PDO cheese	ITAP, HAO-DIMITRA
Pichia fermentans OZ3	Feta PDO cheese	ITAP, HAO-DIMITRA
Pichia fermentans KZ5	Feta PDO cheese	ITAP, HAO-DIMITRA
Pichia fermentans KZ10	Feta PDO cheese	ITAP, HAO-DIMITRA
Pichia fermentans KZ11	Feta PDO cheese	ITAP, HAO-DIMITRA
Pichia fermentans KZ12	Feta PDO cheese	ITAP, HAO-DIMITRA
Candida zaylanoides KZ7	Feta PDO cheese	ITAP, HAO-DIMITRA
Candida zaylanoides KZ8	Feta PDO cheese	ITAP, HAO-DIMITRA
Yarrowia lipolytica OZ5	Feta PDO cheese	ITAP, HAO-DIMITRA

Supplementary Table S2: MIC values and their standard errors from optical density technique (% v/v concentration of Chios mastic gum essential oil in broths using 3 log CFU/mL inoculum level of each microorganism).

**Microorganism**	**MIC**
Listeria monocytogenes FMCC-B-128	0.97±0.05
Listeria monocytogenes FMCC-B-129	1.47±0.15
Listeria monocytogenes FMCC-B-133	0.99±0.10
Listeria monocytogenes DSMZ 15675	1.79±0.02
Listeria monocytogenes DSMZ19094	1.39±0.02
Lactiplantibacillus plantarum T75	1.47±0.01
Leuconostoc mesenteroides FMX3	0.77±0.05
Enterococus faecium SMX4	0.91±0.00
Pichia fermentans OZ1	0.02±0.00
Pichia fermentans OZ3	0.01±0.00
Pichia fermentans KZ5	0.37±0.04
Pichia fermentans KZ10	0.02±0.00
Pichia fermentans KZ11	0.02±0.00
Pichia fermentans KZ12	0.03±0.00
Candida zeylanoides KZ7	0.72±0.01
Candida zeylanoides KZ8	0.55±0.02
Yarrowia lipolytica OZ5	0.008±0.01

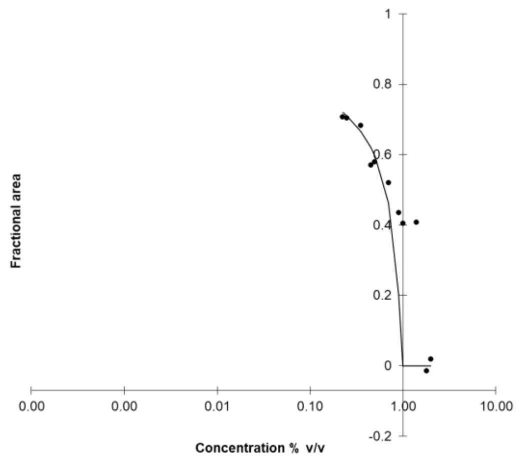

Supplementary Figure S1: The inhibition profile of Chios mastic gum essential oil against *Listeria monocytogenes* FMCC-B-133 with optical density technique. ● Dots represented mean values of 6 replicates.

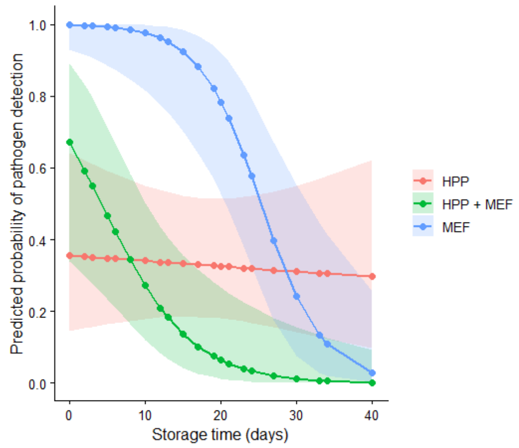

Supplementary Figure S2: Model-predicted probability of *Listeria monocytogenes* detection during storage of cheese slices at 4 °C. Probabilities were estimated using a generalized linear mixed model (binomial distribution, logit link), including treatment, storage time, and their interaction as fixed effects and batch as a random factor. Shaded areas represent 95% confidence intervals. Detection probability incorporates both direct enumeration and enrichment-only positive samples.
